# Automatic snoring detection using a hybrid 1D–2D convolutional neural network

**DOI:** 10.1038/s41598-023-41170-w

**Published:** 2023-08-28

**Authors:** Ruixue Li, Wenjun Li, Keqiang Yue, Rulin Zhang, Yilin Li

**Affiliations:** Key Laboratory of RF Circuits and Systems, Hangzhou Dianzi University, Hangzhou, Zhejiang China

**Keywords:** Biomedical engineering, Respiratory tract diseases, Data mining, Data processing

## Abstract

Snoring, as a prevalent symptom, seriously interferes with life quality of patients with sleep disordered breathing only (simple snorers), patients with obstructive sleep apnea (OSA) and their bed partners. Researches have shown that snoring could be used for screening and diagnosis of OSA. Therefore, accurate detection of snoring sounds from sleep respiratory audio at night has been one of the most important parts. Considered that the snoring is somewhat dangerously overlooked around the world, an automatic and high-precision snoring detection algorithm is required. In this work, we designed a non-contact data acquire equipment to record nocturnal sleep respiratory audio of subjects in their private bedrooms, and proposed a hybrid convolutional neural network (CNN) model for the automatic snore detection. This model consists of a one-dimensional (1D) CNN processing the original signal and a two-dimensional (2D) CNN representing images mapped by the visibility graph method. In our experiment, our algorithm achieves an average classification accuracy of 89.3%, an average sensitivity of 89.7%, an average specificity of 88.5%, and an average AUC of 0.947, which surpasses some state-of-the-art models trained on our data. In conclusion, our results indicate that the proposed method in this study could be effective and significance for massive screening of OSA patients in daily life. And our work provides an alternative framework for time series analysis.

## Introduction

Snoring is a breathing-related event, and it is generated by the vibration of anatomical structures in the upper airway during sleep, such as pharyngeal walls, soft palate, uvula, and tonsils^1^. Epidemiologic studies have shown that habitual snoring affects 9–17% of the worldwide adult population including about 40% of men and 24% of women^2,3^. Snoring disrupts seriously sleep both quantity and quality of snorers and anyone who shares the same sleeping space. Consequently, it often leads to daytime sleepiness and increased inattentiveness elevating the risk of accidents, as well as certain health conditions such as sleep disturbance, systemic arterial hypertension and coronary artery disease^4^. However, due to subjective neglect and limited medical resources, there are approximately 80–90% of OSA population in the developed world remaining undiagnosed and untreated, while the severity of this issue is even more pronounced in the developing world^5,6^.

In general, snoring occurs when the person sleeps on the back. For a snorer without any other nocturnal respiratory pathology (simple snore), it could be relieved naturally by sleeping on the side^7^. So, some gadgets have been developed to prevent snoring by providing some kind of notification to snorers when snoring occurs^8–10^. In addition, as one of the earliest and most prevalent nocturnal symptoms, snoring appears in 70–90% of obstructive sleep apnea (OSA) patients^11^. And studies have shown that the characteristics of snoring sounds, such as intensity^12^, spectral^13^, pitch-related features^14,15^, time and frequency-related parameters^16^, regularity parameters^17^ and some other features^18,19^, could be used to describe the different between simple snorers and OSA patients, as well as the severity of OSA. In addition, compared with the Polysomnography (PSG), the equipment recording audio signal is convenient, low-cost and non-contacting. Therefore, snoring signal has been emerged as one of primary physiological indicators to assess the condition of snorers. As a result, automatic detection of snoring sounds has garnered significant attention in academic research, becoming a prominent area of interest.

In light of the increasing community prevalence of OSA and growing demand for medical care personnel and supplies^20^, researchers have dedicated to achieving automated detection of snoring events with the help of computer technologies over the past two decades^21^. In early time, artificial extraction of physical or mathematical features was performed on the raw audio signal, which were subsequently applied to train classifiers, such as hidden Markov model (HMM) based on 12 Mel-frequency cepstral coefficients (MFCCs)^22^, unsupervised clustering algorithms based on formant frequencies or low-dimensional features by principal component analysis (PCA)^23–25^, AdaBoost classifier based on features in time and spectral domains^26^, quadratic discriminant analysis for formant frequencies^27^, K nearest neighbors (KNN) model trained by MFCCs and empirical mode decomposition^28^. Gaussian mixture model (GMM) trained by 40 features in time, energy and frequency domain^29^, linear regression models based on average normalized energy in subband^30^, support vector machine (SVM) based on multi-features in time domain^31^ and artificial neural network (ANN) band on temporal and spectral features^32,33^. The results have demonstrated the effectiveness of these methods in snoring recognition. However, it remains challenging to determine optimal feature set due to the diversity and nonlinearity of snoring sound.

In order to tackle the challenges of feature extraction, researchers have successfully applied deep learning algorithms, showing remarkable performance in feature representation of images and natural language, to analyze various physiological signals, including electrocardiogram (ECG)^34^, ballistocardiogram (BCG)^35^, vectorcardiography (VCG)^36^, electroencephalograph (EEG)^37^, electromyography (EMG)^38^, among others. And these algorithms have yielded promising results in characterizing the nocturnal sleep respiratory audio signal subsequently. For instance, Nguyen et al.^39^ and Çavuşoğlu et al.^40^ respectively utilized multilayer perceptron neural networks (MLP) to differentiate between snore and non-snore sounds; Arsenali et al.^41^ applied a long-short term memory (LSTM) model to classify snoring and non-snoring sounds after extracting MFCCs; Sun et al.^42^ proposed *SnoreNet,* a one-dimensional CNN (1D CNN) that directly operates on raw sound signals without manually crafted features; Khan et al.^8^ developed a two-dimension CNN (2D CNN) to analyze MFCCs images for automatically detecting snoring and applied it into a wearable gadget; Jiang et al.^43^ found an optional combination of Mel-spectrogram and CNN-LSTM-DNN for snoring recognition; Xie et al.^44^ employed a CNN to extract features from the constant-Q transformation (CQT) spectrogram, and then a recurrent neural network (RNN) was utilized to process the sequential CNN output for classifying the audio signal to snore or non-snore events. The comprehensive experiment settings and results of these researches are provided in Table 1.

**Table 1:** Summary of deep learning algorithms for snoring detection.

References	Year	Dataset/**E**nvironment	Equipment	Features	Classifiers	Results
^39^	2015	15 subjects	A unidirectional mic. faced the neck	Raw audio signal	f-MLP	Accuracy: 94.8%-96.6%
^40^	2017	68 subjects Sleep Studies Laboratory	A mic. placed 30 cm from subject’s mouth	Frequency domain features	MLP	Accuracy: 81.2%
^41^	2018	20 subjects Hospital	A mic. placed 70 cm from the top end of the bed	MFCCs	RNN	Accuracy: 95% Sensitivity: 92% Specificity: 98%
^42^	2019	10 subjects Subject’s private bedroom	A phone placed 50 cm away from subject’s head	Raw audio signal	1D CNN	Average precision: 81.82%
^8^	2019	1000 samples Online source	–	MFCCs	2D CNN	Accuracy: 96%
^43^	2020	15 subjects Hospital	A mic. placed 45 cm above the subject’ s mouth	Mel-spectrogram	CNN–LSTM–DNN	Accuracy: 95.07% Sensitivity: 95.42% Specificity: 95.82%
^44^	2021	38 subjects Sleep Laboratory	A mic. placed 70–130 cm from the top end of the bed	CQT-spectrogram	CNN–LSTM	Accuracy: 95.3% Sensitivity: 92.2% Specificity: 97.7%

Although most of deep learning algorithms performed well in classification of snoring and non-snoring episodes, there still are some drawbacks in these studies. Firstly, the training data was limited in availability and lacked diversity. So far, the data acquisition in most of researches was conducted in sleep laboratory or hospitals, where the sleep environment tends to be relatively quieter compared to a private bedroom at home. It means the signal to noise ratio (SNR) of recorded audio signal might be higher. In a few studies where experiments were performed at home, there only was less than 10 participants^42^. It is negative to train a robust classifier when these data recorded from the same period and a small number of subjects was used. Secondly, a new representation for snoring sound is needed. Some researches have shown that the effectiveness of spectrograms, Mel-spectrum in particular, in snoring detection while almost the same accuracy was obtained for many classifiers including single CNN^8^, LSTM^41^ and hybrid models^43,44^. It could be inferred that one of challenge in snoring detection is to find a better feature representation for raw audio signals at present.

In this study, we put forth several solutions to the aforementioned issues. In order to increase the diversity of samples, we design an experiment involved more than eighty participants and lasted more than ten months. Moreover, the data acquisition in this experiment was conducted at subject’s habitual sleep environment. In addition, the visibility graph (VG) method is introduced to represent nonlinearity of snoring sounds using mapped images. And then, a novel hybrid model combined 1D CNN and 2D CNN architectures is proposed for snoring recognition. Therein, the 1D CNN is used to for raw signal analysis and 2D CNN for corresponding images mapped by VG. And features generated by 1D CNN and 2D CNN are concatenated and analyzed by the next fully connected layer.

The main contributions of this paper are shown as following:

- A larger and more diverse data set of nocturnal sleep respiratory audio signals recorded in subjects’ private homes is built in this work.
- The VG method is first introduced to represent snoring sounds using mapped images.
- A hybrid 1D-2D CNN framework is proposed for snoring sounds recognition, which is more accurate and robust than state-of-the-art deep learning models on our dataset.

The remainder of this paper is organized as follow. “Materials and methods” section describes the corpus of respiratory audios, and presents our processing methods including the visibility graph for data transformation and CNN model for classification in this paper. And then in “Experimental results” section, experimental results show the performance of our model in snoring classification task. The final section of this paper discusses our results and make some concluding remarks.

## Materials and methods

### Data recording

Support by the Affiliated of Hangzhou Normal University (Zhejiang, China), we recorded 88 individuals between 12 and 81 years old including 23 females and 65 males in our experiment from March 2019 until December 2019. All of them or their guardians on their behalf signed informed consent forms prior to our study. In our experiments, all methods met the ethical principles of the Declaration of Helsinki, the guidelines of the relevant guidelines and regulations. And the protocol for this data analysis study was approved by the Zhejiang Natural Sciences Foundation Committee and Ethics Board of Hangzhou Dianzi University.

Based on the aim of our research, a portable PSG and a high-fidelity sound acquisition equipment were used to recording respiratory sounds during overnight sleep in home environment instead of sleep center in studies before. Therein, the sound acquisition equipment was designed by ourselves, including a control module taking the I.MX6ULL as a core, a high-resolution microphone (NIS-80V, FengHuo Electronic Technology Co., Ltd, GuangDong, China; 20–2000 Hz frequency range, − 45 dB sensitivity), a power supply module, and a transmission module allowing online and offline data storage and transmission. During the experiment, it was placed around the subject at a distance in the range of 20–150 cm, and recorded monophonic nocturnal breathing sounds with a sampling frequency of 16,000 Hz. Finally, these signals were saved as some wave format files where each of them was 100 M. On the other hand, the portable PSG recorded various physiological signals including oxygen saturation, sphygmic and respiratory effort at a 10 Hz sampling frequency, which were used to diagnose OSAHS of subjects by a medical professional. In our experiment, the subjects contained simple snorers and OSAHS patients with different severity. Detail information of subjects are summarized in Table 2. The average duration of a respiratory sound recorded was 7 h and 26 min.

**Table 2:** Anthropometric information of subjects in our experiment.

Features	Normal	Mild	Moderate	Severe
No. of subjects	20	15	21	22
Age	35.10 ± 12.51	44.91 ± 15.50	49.22 ± 11.29	48.13 ± 14.01
BMI	24.43 ± 3.52	25.21 ± 3.38	26.90 ± 3.13	29.38 ± 9.15
AHI	2.00 ± 1.05	8.35 ± 2.56	22.47 ± 2.89	46.89 ± 15.77

The long breathing sound signal recorded by microphone captures both snoring events and normal respiratory sound. First, some alternative sound episodes were extracted from the raw respiratory sounds by a clustering algorithm^45^. And then, these sound episodes were manually annotated as snore or non-snore in visual and auditory inspection. In order to ensure the diversity of snoring, part of subjects from normal snorers, mild, moderate, and severe groups were chosen. In total, 5441 sound episodes including 3384 snoring segments and 2057 non-snore ones were chosen in our experiment. The average duration of snoring episodes is approximately 1000 ms, and non-snore one is about 3000 ms in duration.

### Preprocessing

**Figure 1:**
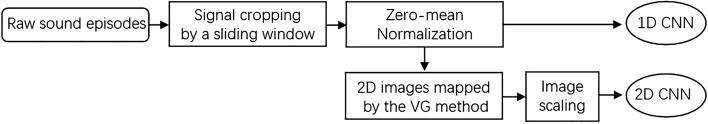
The flow chart and methods included in the preprocessing stage.

In this paper, there are several preprocessing tasks performed for preparation. The methods involved and workflow of data processing is shown in Fig. 1.

#### Audio signal cropping

The audio signals in our database had various duration. It is a challenge in audio processing using the CNN for which the size of the input sample must be fixed and consistent. One of the most common methods to achieve this requirement of the CNN input layer is to split the audio signal into fixed-length fragments with the help of a sliding window.

**Figure 2:**
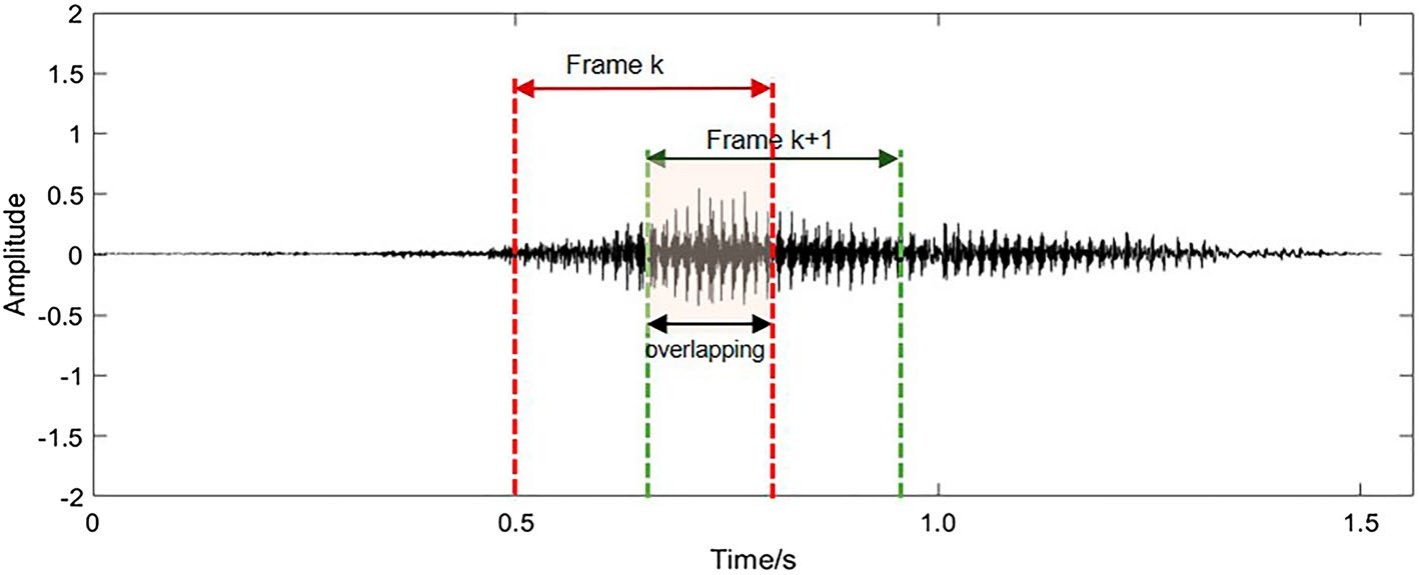
The process of framing audio signals into several sound segments with a half of overlapping.

For audio signals recorded in our experiments, we used a time window with appropriate width to capture continuous sound segments. In order to reserve information as much as possible, adjacent time windows may have a certain percentage of overlapping. The process of framing audio signals into frames was illustrated in Fig. 2. It is worth noting that the overlapping ratio of time windows varied across different groups of audio signals to prevent potential issues of data imbalance^45^ in our dataset. Meanwhile, the shorter frame of audio signal could keep the CNN model compact. In this study, based on the minimum duration of a snoring segment, length of the time window is set to be 0.3 s. And then, these sound clips were normalized by z-score standardization for improving precision and convergence speed of our deep learning model.

#### Visibility graph method

The visibility graph (VG) method, proposed by Lacasa et al.^46^, converts a time series into a graph based on a geometric principle of visibility. Researches have shown that the mapped visibility graph inherits several properties of the series in its structure: the periodic series is converted into regular graph, random series into random graphs and fractional series into scale-free graph^46,47^. So, it has been widely used to analyze time series in different fields including psychology, physics, medicine and economics^48–51^. In this paper, we regard the associated graph of time series as an image, which enables CNN-based analysis.

The criterion of image mapping by VG is established as follow: any arbitrary two data value $(t_{a} ,x_{a} )$ and $(t_{b} ,x_{b} )$ in the time series $\{ t_{i} ,x_{i} \} (i{ = 1,2,} \cdots )$ will have visibility, and pixel value is 1 in corresponding position $(t_{a} ,t_{b} )$ of the mapped image, if any other data $(t_{c} ,x_{c} )$ with $t_{a} < t_{c} < t_{b}$ satisfies

1 $$ x_{c} < x_{a} - (x_{b} - x_{a} )\frac{{t_{c} - t_{a} }}{{t_{b} - t_{a} }}. $$

On the basis of this principle, the mapped image is a binary matrix where the value of corresponding element is 1 if two nodes is visible, otherwise it is 0. That is, the fluctuation of time series in time domain could be transformed as various geometric graph in time-time space. It provides a new perspective to explore complex dynamics of series more intuitively. In our study, each of sound clips were mapped into images with resolution of 4800 × 4800. Before transferred into the CNN model, these images were resized to 256 × 256 for simplified calculation.

### Convolutional neural networks

**Figure 3:**
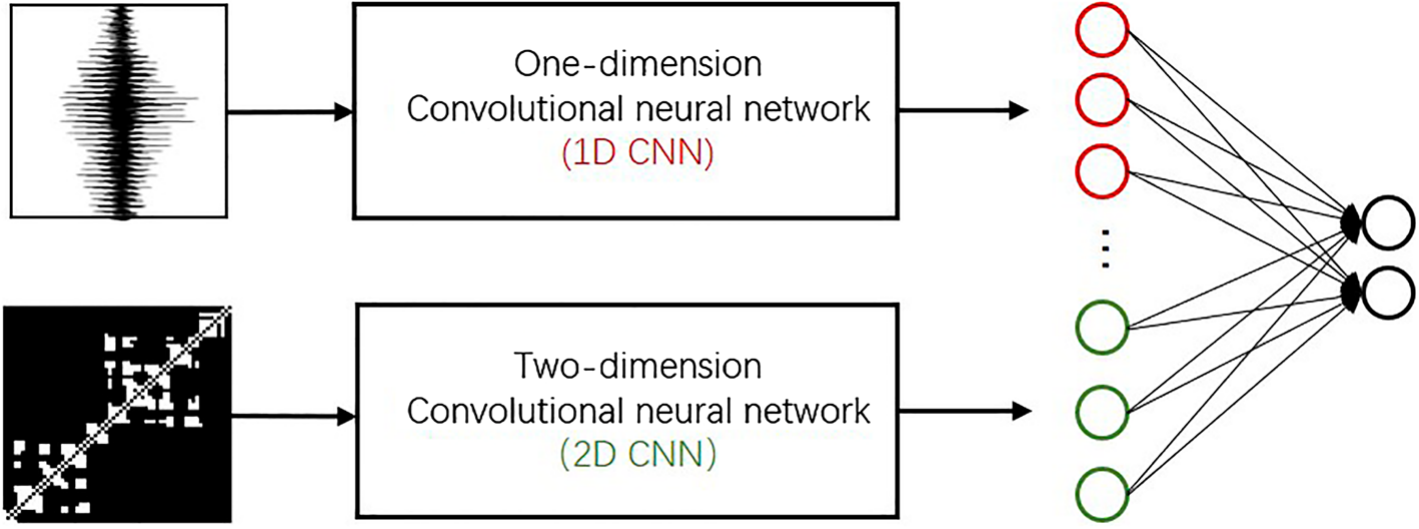
A framework of our model proposed in this study. The 1D CNN is used to present raw audio signals, 2D CNN is used to analyze mapped images, and two fully connected layers is a classifier.

For the sound segment and its corresponding visibility graph, a deep neural network combined 1D CNN and 2D CNN was proposed to represent their characteristics, respectively. And then two fully connected layers were used to recognize these united features as snore or non-snore sound. The framework is shown in Fig. 3.

#### 1D CNN topology

**Figure 4:**
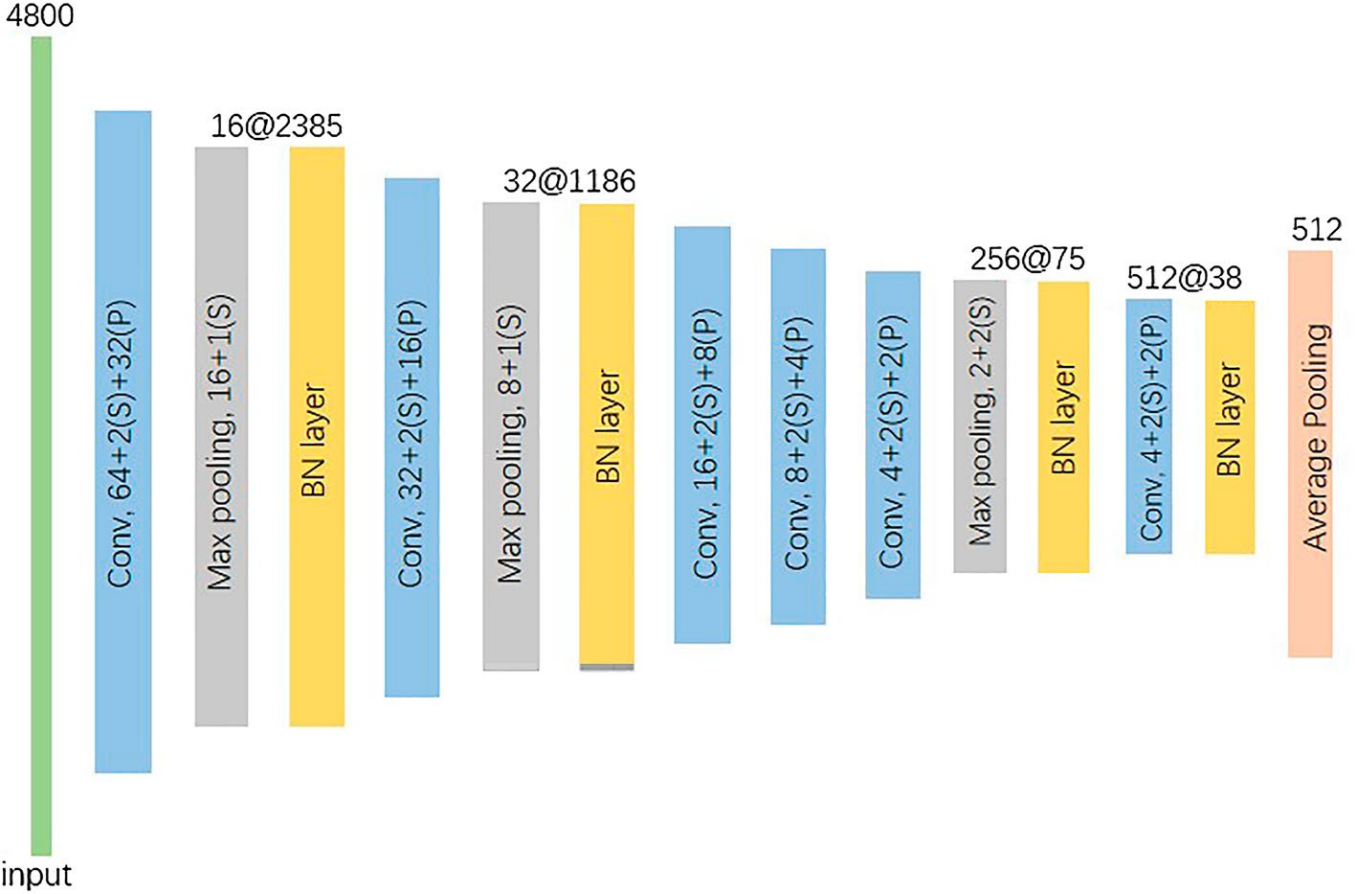
The architecture of the 1D CNN for representation of raw audio waveform.

For the raw audio signals, a compact 1D CNN architecture with a reduced number of parameters is built in this paper as shown in Fig. 4. It is based on a 1D CNN architecture for environmental sound classification^52^. There are four trainable convolutional layers, interlaced with batch normalization layers^53^ and max pooling layers in this architecture. At last, the output of the last batch normalization layer is flattened by an average pooling layer, and they will be concatenated with features extracted by 2D CNN. Based on the assumption that the first layer in reflex arc has a more global view of the audio signal, the kernel size (called receptive field) in first convolutional layer is set to 1 × 64, and then decreases progressively to 1 × 8 for the next three convolutional layers. This architecture has been verified it is powerful enough to extract relevant low-level and high-level information from the small audio data set^52^.

#### 2D CNN topology

**Figure 5:**
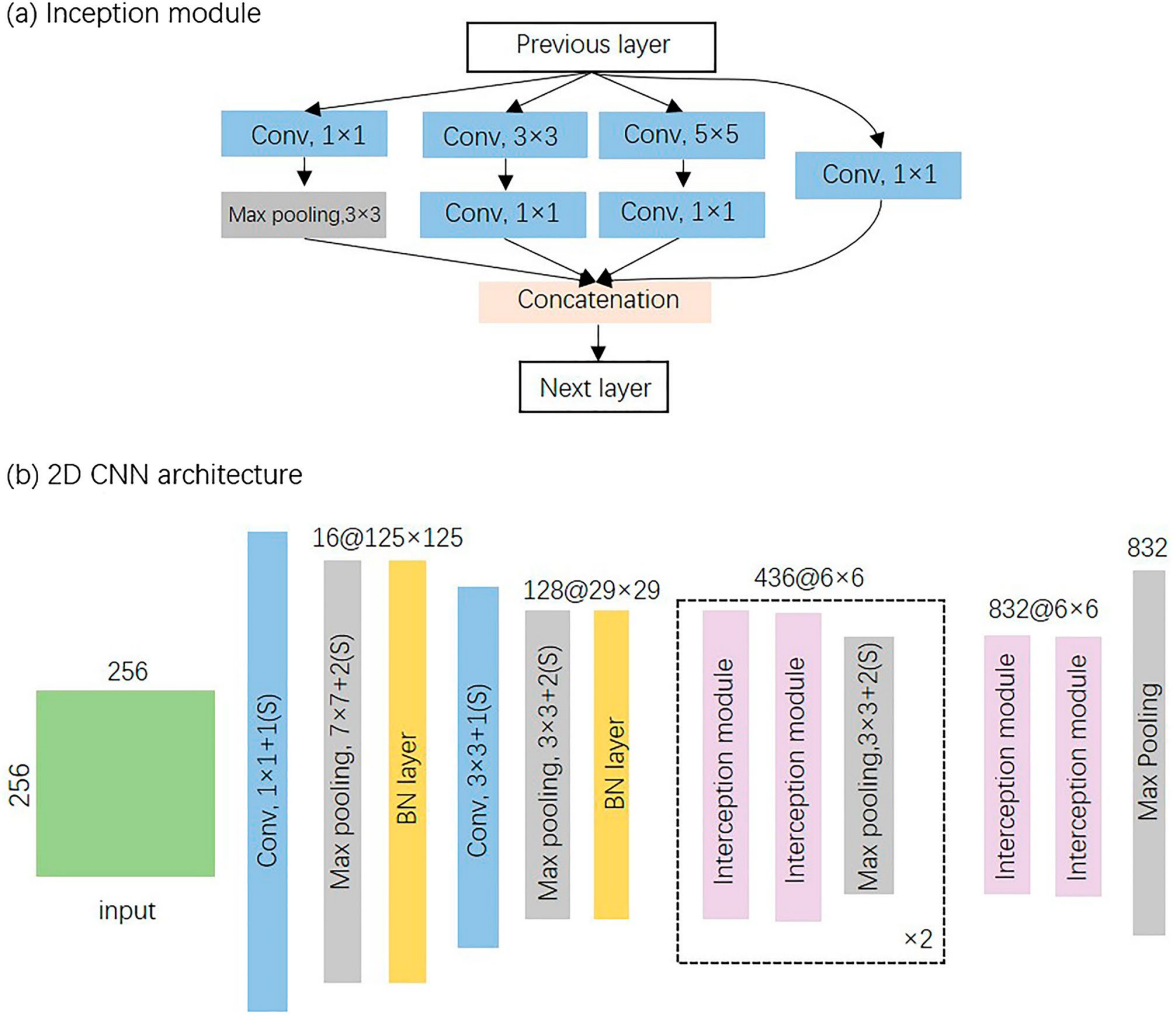
The architecture of the 2D CNN for representation of mapped visibility graph.

So far, there have been lots of mature 2D CNN models widely used and done well in computer vision (CV) and natural language processing (NLP). Considering the sparse characteristics and multi-scale information carried by those nested quadrates of the mapped images generated by the VG method, we introduced an inception module in our 2D CNN model for snoring sound recognition^54^, as shown in Fig. 5a. The parallel multiscale convolutional layers could keep the computational budget constant while increasing the depth and width of the CNN models. It is helpful to overcome some problems such as computational expensiveness, over-fitting and vanishing gradient^54^. In our 2D CNN described by Fig. 5b, there are three convolutional layers, six inception modules and some pooling layers, which takes responsibility of feature representation. At last, two fully connected layers are applied to classify the concatenated features as snore or non-snore sounds.

In our model, the ReLU is chosen as the activation function for all convolutional layers, the ADAM algorithm and a binary cross entropy loss are applied to train optimal model based on error back propagation algorithm. In order to prevent over-fitting during training, we try our best to keep the CNN architecture simple and shallow as far as possible, L_1_, L_2_-norm with penalty coefficients of 0.001 were added to the loss function, and the dropout algorithm was applied to train our model. The learning rate had a 20-step gradual warmup from an initial value of 1e-6, and then it had a step-based decrement.

### Postprocessing

**Figure 6:**
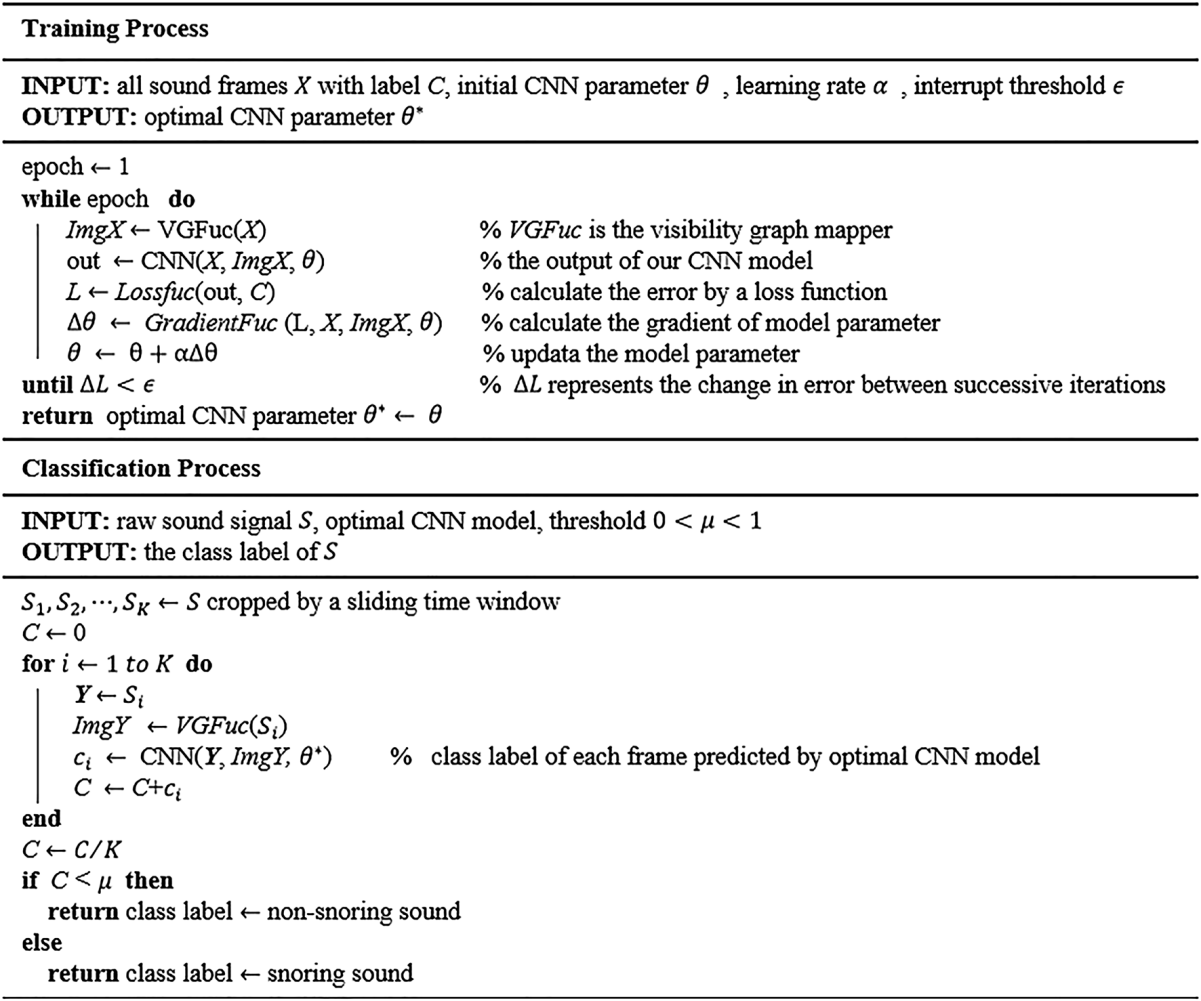
The pseudocode of the proposed 1D-2D CNN algorithm.

In our database, there are a minimum of one sound frames and a maximum of about twenty frames extracted from a typical sound signal. And our CNN model is applied to process each sound clip. Therefore, for a sound event $S$, its label $C$ is decided by aggregating the CNN predictions of all split frames $S_{1} ,S_{2} , \ldots ,S_{K}$ described as

2 $$ C = \left\{ {\begin{array}{*{20}l} {0,} \hfill & {\sum\nolimits_{i = 1}^{K} {c_{i} /K < 0.5} } \hfill \\ {1,} \hfill & {else} \hfill \\ \end{array} } \right. $$

where $c_{i}$ is the prediction for the frame $S_{i}$ ($i = 1,2, \ldots ,K$), which value is taken as 0 or 1 representing non-snore class and snore class respectively. The pseudocode of our 1D-2D CNN algorithm is given in Fig. 6.

### System evaluation

The detection performance of our model is evaluated by confusion matrix. It described how many results were correctly classified and how many were incorrectly classified for each of categories. Further, accuracy, sensitivity, and specificity are calculated based on Eq. (3). Therein, accuracy evaluates the recognition capability overall of our model, which is a common assessment criterion in classification tasks. Sensitivity, also called as recall ratio, is the ratio of correct positive predictions in all true samples, and specificity is the ratio of correct negative predictions in all false ones. Both of them were supplemented to profile our model in more detail. In clinical medicine, the former is of great importance for preliminary screening of diseases and the later for their diagnosis. The possible values of above their indexes are from 0 to 1, and the higher their values, the better the performance of our classifier

3 $$ \begin{aligned} & Accuracy = \frac{{N_{TP} + N_{FN} }}{{N_{TP} + N_{TN} + N_{FP} + N_{FN} }} \\ & Sensitivity = \frac{{N_{TP} }}{{N_{TP} + N_{FN} }} \\ & Specificity = \frac{{N_{TN} }}{{N_{TN} + N_{FP} }} \\ \end{aligned} $$

where $N_{TP,TN,FP,FN}$ represents the number of true positive (*TP*), true negative (*TN*), false positive (*FP*) and false negative (*FN*).

In addition, classification scores were obtained from a receiver-operating curve (ROC) and the area under this curve (AUC). The ROC is determined from the false positive rate and the true positive rates after applying different thresholds. The AUC score of 1.0 depicts perfect predictions, while a random classifier achieves an AUC score of 0.5. A higher AUC score means a better predictor. The characteristic of ROC and AUC is that their shape and score remain constant as the distribution of positive and negative samples while the samples change. So, it was a more objective index to assess our model performance.

## Experimental results

We use the fivefold cross-validation strategy to train and test the proposed model. The 88 subjects were randomly divided into five groups including three eighteen-subject groups and two seventeen-subject groups. In each trial, four groups were used for training, while the remaining group was reserved for testing purposes. By ensuring independence at the subject level, we mitigate the risk of overvaluation caused by data from the same subject is both in training and testing sets.

Each snoring/non-snoring episode was divided into multiple fragments by methods in “Data recording” section. And its predictive label was decided by model outputs of all fragments based on Eq. (2). The performance of our model was evaluated by confusion matrices, indexes described in Eq. (3), and AUC. All preprocessing and analysis were performed using MATLAB R2019b (The MathWorks, Inc., Natick, MA, USA). The deep neural network, as shown in Figs. 3, 4 and 5, was constructed in Python 3.8 using Pytorch library 1.10. The model was trained on a desktop computer with Intel Core i9 (8 Cores) 3.5 GHz microprocessor, 64 GB BAM and NVIDIA GeForce RTX 3090 graphics processing unit (GPU).

**Figure 7:**
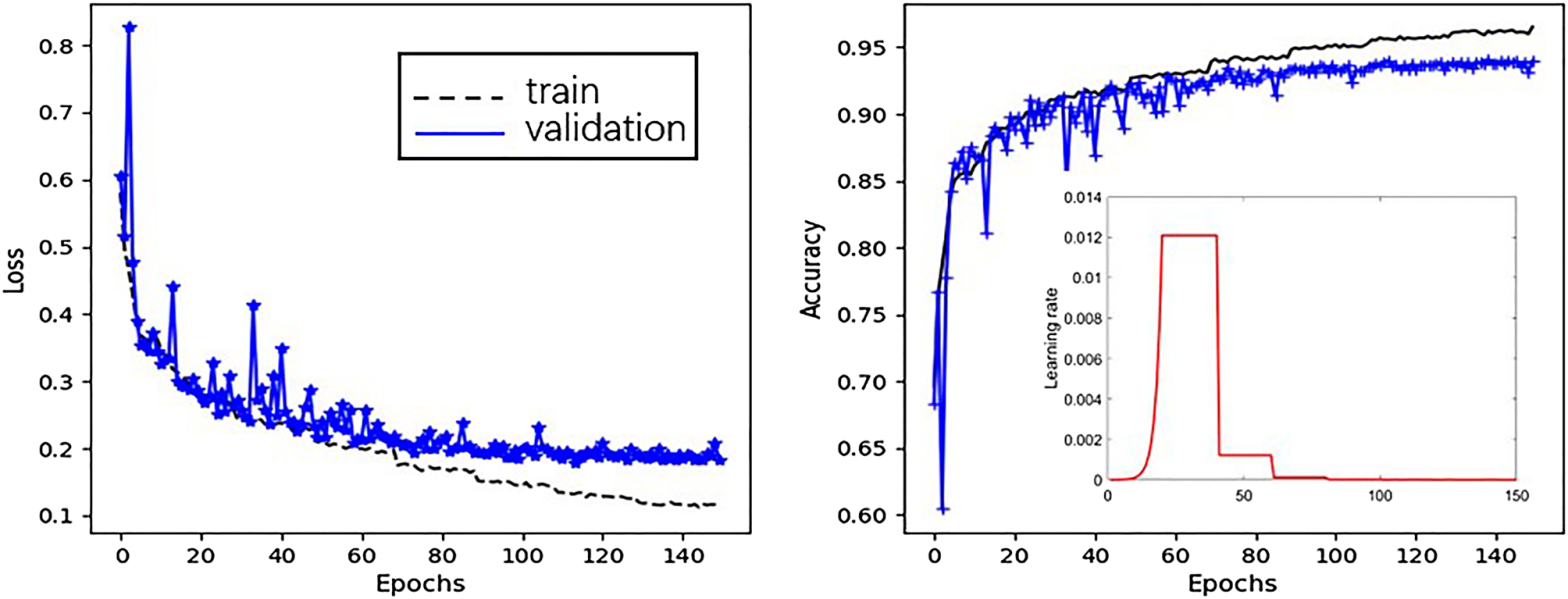
The average accuracy and loss for snoring fragments recognition in our experiment.

In our experiment, a batch size of 150 samples was used for training our CNN model and it was trained up to 150 epochs. The learning rate followed a 20-step gradual warm-up starting from an initial value of 1e−6 at a rate of 1.6. Afterward, it was reduced to 10% of its original magnitude every 20 epochs. Figure 7 shows the variation of loss and accuracy of our model for snoring fragments recognition in a trail. It is obvious that our CNN model is converged gradually with the increasing of iterations and reaches stability after 70 iterations. In order to avoid over-fitting, the training was halted at 90 steps during iteration. After postprocessing, the results of the fivefold cross-validation for all 88 subjects are presented by confusion matrices in Table 3 and the calculated performance indices in Table 4. In our experiment, we achieved accuracy ranging from 86.4 to 91.2%, sensitivity between 88.1 and 91.6%, specificity between 83.0 and 91.8%, and AUC between 0.908 and 0.973.

**Table 3:** Confusion matrix of snore detection using fivefold cross-validation.

Trials			Ground truth	
			Snore	Non-snore
Model prediction	1	Snore	604	54
		Non-snore	40	356
	2	Snore	572	77
		Non-snore	56	274
	3	Snore	806	74
		Non-snore	34	327
	4	Snore	632	76
		Non-snore	32	362
	5	Snore	553	56
		Non-snore	51	343

**Table 4:** Performance indices of our model in each trail and average performance.

Trials	Accuracy (%)	Sensitivity (%)	Specificity (%)	AUC
1	89.3	88.9	90.0	0.951
2	86.4	88.1	83.0	0.908
3	91.2	91.6	90.7	0.973
4	90.2	89.2	91.8	0.956
5	89.3	90.8	87.1	0.946

Here, two snoring detection models including two proposed state-of-the-art models^8,44^ were chosen for comparison. And they were retained and their hyper-parameters were retuned based on our sound samples at subject level. At last, the results of these models on our dataset are summarized by Table 5, where the mean and standard deviation of indexes of all five runs are shown. All of these deep neural network algorithms extracted features automatically using CNN layers from raw audio signals or their transformations. Compared with datasets in these previous studies, our data is more diverse and lower signal-to-noise ratio at the case of little number of the event sounds. Affected by that, these three models trained by our data had poorer performances than that trained by their own dataset. By contrast, our method achieved better performances in terms of accuracy, sensitivity, specificity and AUC. And results show that the standard deviations of index values in five trials are smaller than other methods, which could be suggested that our algorithm is more robust. In addition, we trained a single 1D CNN with a same architecture shown in Fig. 4, and obtained a poor performance than our hybrid 1D-2D CNN model for snoring recognition. It could be inferred that images transformed by VG method provide some additional information for snore recognition.

**Table 5:** Comparison to baseline models on our dataset.

Models	Accuracy (%)	Sensitivity (%)	Specificity (%)	AUC
2D CNN^8^	84.7 ± 4.5	90.3 ± 7.1	66.9 ± 28.9	0.839 ± 0.091
CNN-LSTM^44^	86.3 ± 3.2	90.7 ± 2.1	76.3 ± 8.0	0.908 ± 0.020
1D CNN	85.2 ± 7.6	89.5 ± 8.3	70.5 ± 16.0	0.870 ± 0.062
Our work	89.3 ± 1.8	89.7 ± 1.4	88.5 ± 3.5	0.947 ± 0.024

## Conclusion and discussion

In this paper, we build an audio database recorded by non-contact microphone in subject’s private bedroom and propose a new snoring detection algorithm based on the audio signal and a series of convolutional neural networks. Instead of manually engineered features, the proposed CNN learned information from the raw audio waveform and their VG maps, and was trained by audio data from 88 subjects. In our experiment, our algorithm achieves an average accuracy of 89.3%, which is higher than the accuracy obtained by two state-of-the-art methods in our dataset.

In addition, the VG maps transformed from the audio signal is first applied to snoring classification. The VG method can map the “visibility” relationship between features at different time points in time domain into real value at a particular location in two-dimensional space, and then the change of this relationship is visualized as specific geometrical shape. Experimental results shown in Table 5 indicate that the VG maps might provide higher-order and non-linear information as supplementary of raw audio signals. It is suggested that the VG maps could be an alternative method for time series classification.

Despite the superior performance of the proposed model compared to other state-of-the-art models on our dataset, it is not without its limitations. On one hand, the input size of model might be variable for different tasks. As a hyper-parameter, it should be chosen to balance the amount of information and computation. On the other hand, we assess the models' complexity in snoring detection by calculating the number of parameters and floating-point operations per second (FLOPS). The corresponding results are presented in Table 6. It is evident that the proposed model in this study exhibits a higher parameter count and FLOPS, indicating a greater demand for computational resources. And it is worth noting that this increased complexity does not necessarily confer an advantage in algorithm localization.

**Table 6:** Measurement of models’ complexity on our dataset.

Metrics	2D CNN^8^	CNN–LSTM^44^	1D CNN	Our model
Parameters (M)	1.28	0.25	0.77	3.15
FLOPS (G)	0.17	0.08	0.25	1.57

In addition, there are also shortcomings in our experimental settings. First, studies have shown that the sound characteristics of rare expiration snore sounds are different from the major dominant inspiration^28,55^. But we did not consider the phase of the respiratory cycle in which snore event occurs in our experiment. It makes it difficult for our method to recognize infrequency expiration snore sound. Secondly, the diversity of snore sounds due to different places of pharyngeal constriction^56^ is not taken into account in our study. If some kind of snore sounds is not involved in training, it could be missed by our algorithm.

Therefore, in future work, we will improve further the robustness and generalization of the current snore detection algorithm by adding more expiration snore sounds and considering more snore events due to distinct places of pharyngeal constriction. Furthermore, the original VG method could be replaced by other methods in VG family, which includes HVG, limited penetrable visibility graph (LPVG) and so on, for improvement of noise resistance and computation efficiency^57^. In addition, the VG map used in this study only contains the 0 and 1 elements representing whether the features in different points are visible. It could be improved by depicting degree of visibility between time points to inherit more information from raw time series, which might be meaningful for simplification of snore recognition.

## Data Availability

The data that support the findings of this study are available from The Affiliated Hospital of Hangzhou Normal University, but restrictions apply to the availability of these data, which were used under license for the current study, and so are not publicly available. Data are however available from the authors upon reasonable request and with permission of The Affiliated Hospital of Hangzhou Normal University.
